# Anti-N-Methyl-D-Aspartate Receptor Encephalopathy in a Young Female

**DOI:** 10.7759/cureus.57971

**Published:** 2024-04-10

**Authors:** Ali Ghorbani, Nicholas R Munoz, Syed Ahmed, Salma Yasin, Sophia Ho, Aida Ghorbani, Kurosh Zamiri

**Affiliations:** 1 Internal Medicine, Southwest Healthcare, Temecula, USA; 2 Internal Medicine, Temecula Valley Hospital, Temecula, USA; 3 Neurology, University of California, Los Angeles School of Medicine, Los Angeles, USA; 4 Biology, University of California, Los Angeles School of Medicine, Los Angeles, USA

**Keywords:** nmdar encephalitis, autoantibody behavioral change, nmdar antibody encephalitis, subacute psychosis, acute psychosis, acute encephalitis, anti-nmdar encephalitis

## Abstract

Widely distributed in the central nervous system (CNS), N-methyl-D-aspartate receptors (NMDARs) are believed to be involved in long-term potentiation, essential in regulating and forming memory. This condition primarily occurs in young females because of autoantibodies forming against the N-methyl-D-aspartate receptor-1 (NR1) or N-methyl-D-aspartate receptor-2 (NR2) subunits of NMDAR in the CNS, ultimately portraying a unique psychoneurological phenomenon. Patients with antibodies against NMDAR present with a combination of neurological and psychiatric signs and symptoms. This article presents a case of a young female with no significant past medical, psychological, or surgical history. While being previously diagnosed with acute psychosis, upon arrival at the emergency department (ED), she also displayed an acute decline in judgment, hallucinations, severe agitation, and peculiar behavior, prompting family members to seek medical attention. Consequently, she was evaluated for metabolic and infectious encephalopathy. Following a thorough examination and extensive laboratory imaging, the patient was found to have NMDAR antibody encephalopathy. After dedicated treatment, her two-month follow-up presented a complete resolution of symptoms.

## Introduction

Epidemiologically, anti-N-methyl-D-aspartate receptor (NMDAR) encephalitis is more common than any individual viral encephalitis [1]. This entity was first described in 2005, and its autoantigens were discovered by Dalmau et al. (2007) [1] in a cohort of 12 female patients in the age range of 14-44 years, most of whom had ovarian teratomas, which have been shown to have a strong association with the disease [2]. This autoimmune encephalitis syndrome is a fairly new diagnosis and has been overlooked in the emergency department (ED) due to a lack of symptoms and mechanistic recognition [3].

Anti-NMDAR encephalitis has a higher incidence among females (75%) at younger ages, but after the age of 45, the male-to-female ratio is more balanced [1,2]. The role of estrogen or progesterone in this pathology has not been established. In this case report, we discuss the pathogenic mechanisms and immunologic triggers of anti-NMDAR encephalitis and provide an overview of the treatment and prognosis of this disorder, with a specific focus on the management of common symptoms, complications, and prognosis.

## Case presentation

The patient is a 32-year-old female with no significant past medical history who presented to the emergency department (ED) for acute confusion with an onset of one week. Per her family, the patient was asking repetitive questions, having memory issues, and becoming increasingly paranoid. She was reportedly asking her family to help her as she believed someone was looking for her.

According to the family at the bedside, the patient had been acting progressively odd for a week. She worked as a nanny and usually took a taxi home, but on the day of admission, she could not remember where she lived. The family reported that she occasionally hallucinated that someone was in her room at night. At times, the patient would inappropriately run outside, causing the family to lock the door for the patient's safety. On the day of admission, she became agitated, prompting the family to take the patient to the hospital. Her mother reported that the patient started on a new nasal spray a day prior. Otherwise, the patient's family denied other new medications, illicit drug use, changes in living situation, or any recent travel. They stated that her paternal aunt had a history of similar symptoms, with paranoia and agitation for a short period, which was improved with medication, although they were unable to recall the diagnosis.

The patient's physical examination was remarkable for blood pressure of 98/58 mmHg, heart rate of 78 beats/minute, respiratory rate of 16 breaths/minute, body temperature of 97.8°F, oxygen saturation of 98% on room air, and body mass index of 23.6 kg/m^2^.

She had a blunted affect with slow but fluent speech and was able to answer questions appropriately. She also showed increasing paranoia, stating "Can you please tell the man to not come in or look at me?" Neurologically, the patient followed commands without any focal neurological deficits. Comprehension appeared to be intact. Cerebellar tests including finger-to-nose testing were normal. Cranial nerves II-XII were intact. Muscle strength was normal and sensation intact. She was oriented to year and name, but not situation. The remaining examination, including lungs, cardiovascular, abdomen, extremities, dermatology, and musculoskeletal, was unremarkable.

The patient underwent extensive workup including serology, thyroid-stimulating hormone (TSH), liver function tests (LFTs), rapid plasma reagin (RPR), human immunodeficiency virus (HIV), vitamin B12, serum copper and ceruloplasmin, antinuclear antibody (ANA), erythrocyte sedimentation rate (ESR), C-reactive protein (CRP), and urine toxicology, which were all within normal limits. A diagnostic lumbar puncture (LP) was performed to evaluate cerebrospinal fluid (CSF) for cell count, protein, glucose, culture, and encephalitis panel including anti-NMDAR antibodies, anti-voltage-gated potassium channel antibodies, and anti-glutamic acid decarboxylase antibodies. The patient's laboratory test results are shown in Table 1.

**Table 1:** Laboratory findings of the patient µL: microliter, g/dL: gram per deciliter, fL: femtoliter, pg: picogram, MCV: mean corpuscular volume, MCH: mean corpuscular hemoglobin, MCHC: mean corpuscular hemoglobin concentration, RDW-CV: red cell distribution width-coefficient of variation, eGFR: estimated glomerular filtration rate, ESR: erythrocyte sedimentation rate, mg/dL: milligrams per deciliter, mmol/L: millimole per liter, g/dL: gram per deciliter, IU/L: international unit per liter, mL/min: milliliter per minute, mOsmol/kg: millimole per kilogram, µU/mL: microunit per milliliter, mm/h: millimeter per hour

Laboratory investigation	Patient's laboratory value	Reference range
White blood cell	9.8 × 10^3^/µL	3.4-10.8 × 10^3^/µL
Red blood cell	3.87 × 10^3^/µL	3.77-5.28 × 10^3^/µL
Hemoglobin	12.3 g/dL	11.1-15.9 g/dL
Hematocrit	36.5%	34%-46.6%
MCV	91.3 fL	79-97 fL
MCH	30.1 pg	26.6-33 pg
MCHC	32.8 g/dL	31.5-35.7 g/dL
RDW-CV	14.5%	11.7%-15.4%
Platelet	223 × 10^3^/µL	150-450 × 10^3^/µL
Glucose	112 mg/dL	70-99 mg/dL
Sodium	137 mmol/L	134-144 mmol/L
Potassium	3.9 mmol/L	3.5-5.2 mmol/L
Chloride	102 mmol/L	96-106 mmol/L
CO2	25 mmol/L	20-29 mmol/L
Anion gap	10	8-12
Blood urea nitrogen	19 mg/dL	6-24 mg/dL
Creatinine	0.4 mg/dL	0.57-1 mg/dL
Calcium	8.8 mg/dL	8.7-10.2 mg/dL
Total protein	5.4 g/dL	6-8.5 g/dL
Albumin	3.2 g/dL	3.9-4.9 g/dL
Albumin/globulin ratio	1.45	1.2-2.2
Total bilirubin	0.8 mg/dL	0-1.2 mg/dL
Alkaline phosphatase	112 IU/L	44-121 IU/L
Aspartate aminotransferase	13 IU/L	0-40 IU/L
Alanine aminotransferase	27 IU/L	0-32 IU/L
Estimated creatinine clearance	136.52 mL/min/1.73 m^2^	>90 mL/min/1.73 m^2^
eGFR	>90 mL/min/1.73 m^2^	>59 mL/min/1.73 m^2^
Calculated osmolality	279 mOsmol/kg	275-295 mOsmol/kg
Thyroid-stimulating hormone	1.2 µU/mL	0.450-4.500 µU/mL
Free T4	1.01 ng/dL	0.82-1.77 ng/dL
Rapid plasma reagin	Negative	Negative
ESR	32 mm/h	≤20 mm/h
C-reactive protein	<0.3 mg/dL	<0.3 mg/dL

Laboratory results for beta-human chorionic gonadotropin (β-HCG), rapid plasma reagin (PRP), human immunodeficiency virus (HIV), vitamin B12, serum copper and ceruloplasmin, antinuclear antibody (ANA), and urine toxicology were all unremarkable.

Fluoroscopy-guided lumbar puncture (LP) was performed by an interventional radiologist (IR), and the results are shown in Table 2.

**Table 2:** Cerebrospinal fluid laboratory results of the patient CmH2O: centimeter water, mg/dL: milligram per deciliter, mm^3^: millimeter cube, NMDAR-Ab: N-methyl-D-aspartate receptor antibody, CSF: cerebrospinal fluid, RBC: red blood cell, WBC: white blood cell

CSF analysis	Patient's value	Reference range
Opening pressure	19 Cm/H2O	10-25 CmH2O
Protein content	34 mg/dL	15-60 mg/dL
Glucose level	69 mg/dL	50-80 mg/dL in non-diabetics
Total RBC	4 RBC/mm^3^	0 RBC/mm^3^
Total WBC	37 WBC/mm^3^	0-5 WBC/mm^3^
Neutrophils	4%	0%
Monocyte	2%	30%
Lymphocyte	94%	70%
Syphilis	Negative	Negative
Encephalitis viral panel	Negative	Negative
Autoimmune encephalitis panel	Highly positive for NMDAR-Ab	Negative
NMDAR-Ab titer	1:80	Negative

**Figure 1:**
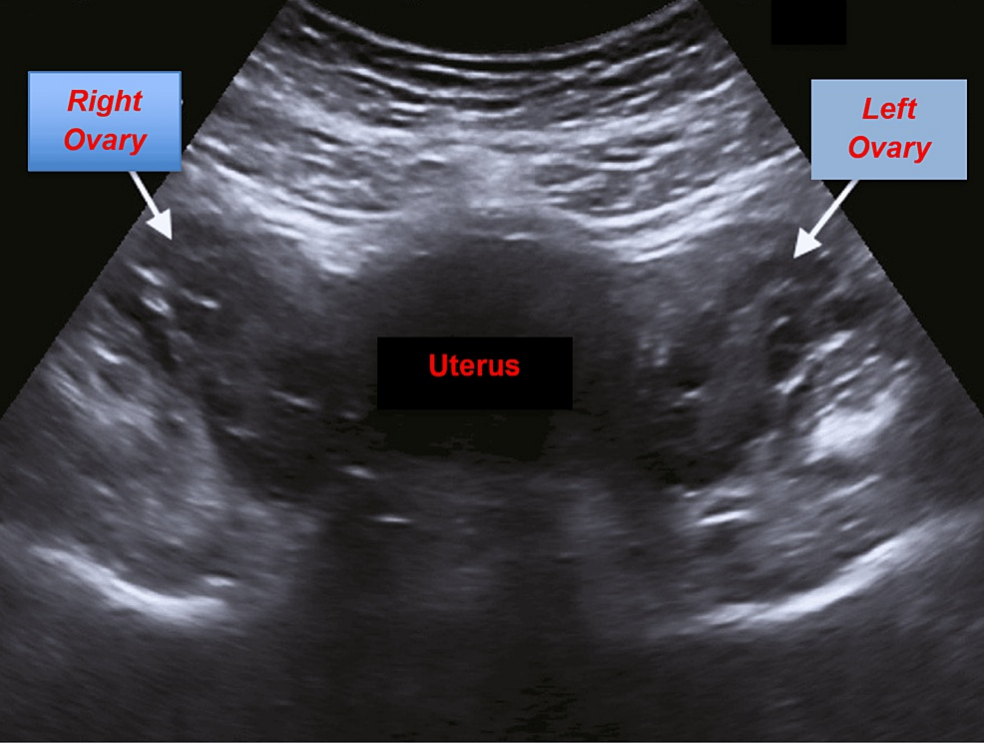
Ultrasound of the pelvis negative for any ovarian mass or cyst

**Figure 2:**
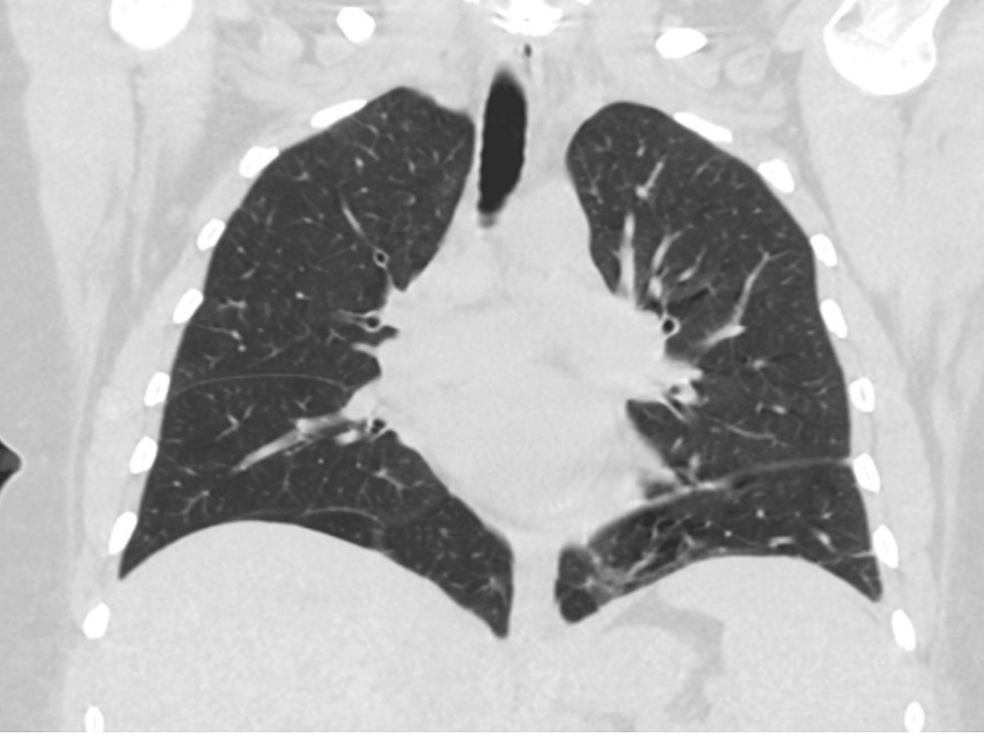
CT of the chest without any pathology CT: computed tomography

**Figure 3:**
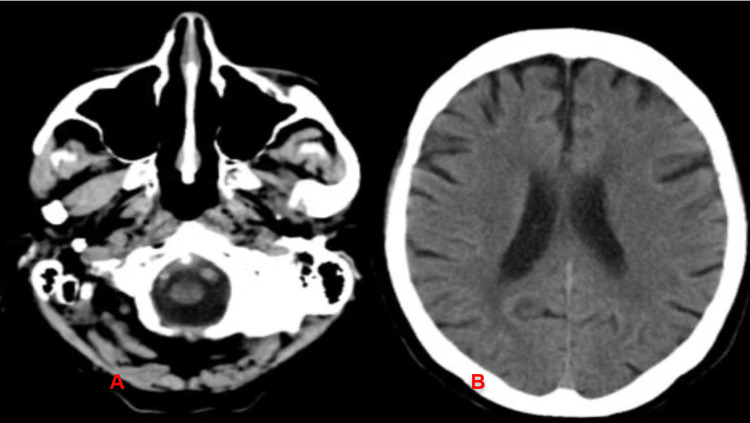
CT of the head without contrast showed no pathology (at rhombencephalon (A) and prosencephalon (B) level) CT of the head without contrast showed no acute intracranial pathology, no signs of bleeding, and no mass effect. CT: computed tomography

**Figure 4:**
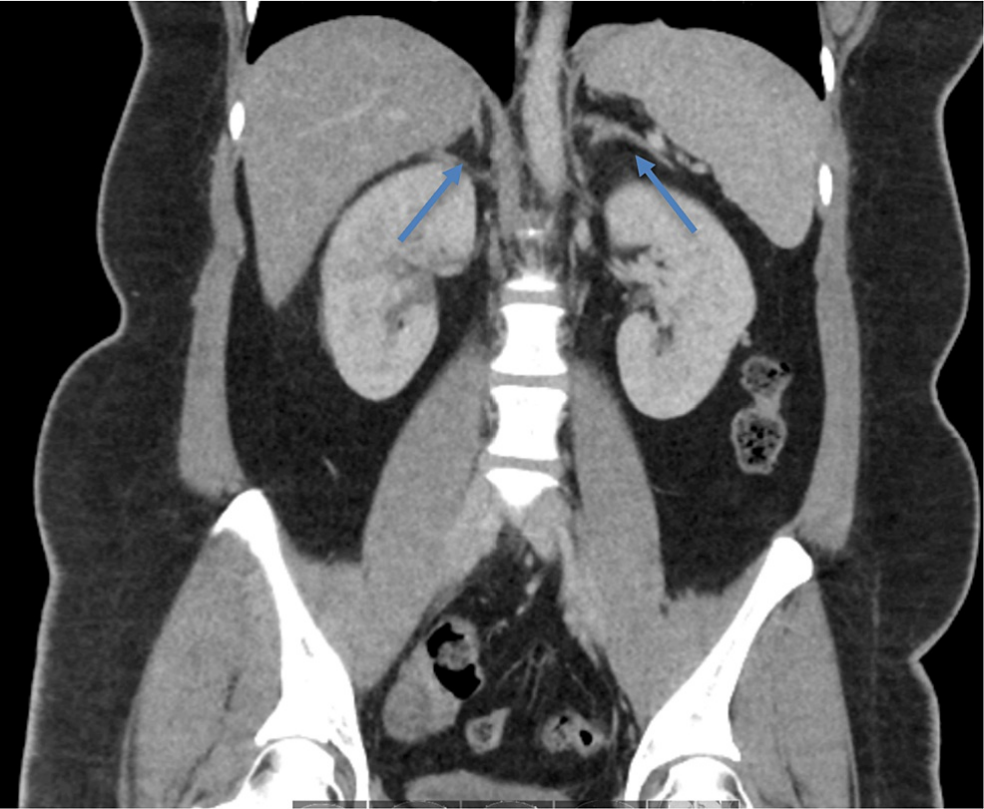
CT of the abdomen negative for any pathology or mass Arrows point to adrenal glands bilaterally. CT: computed tomography

**Figure 5:**
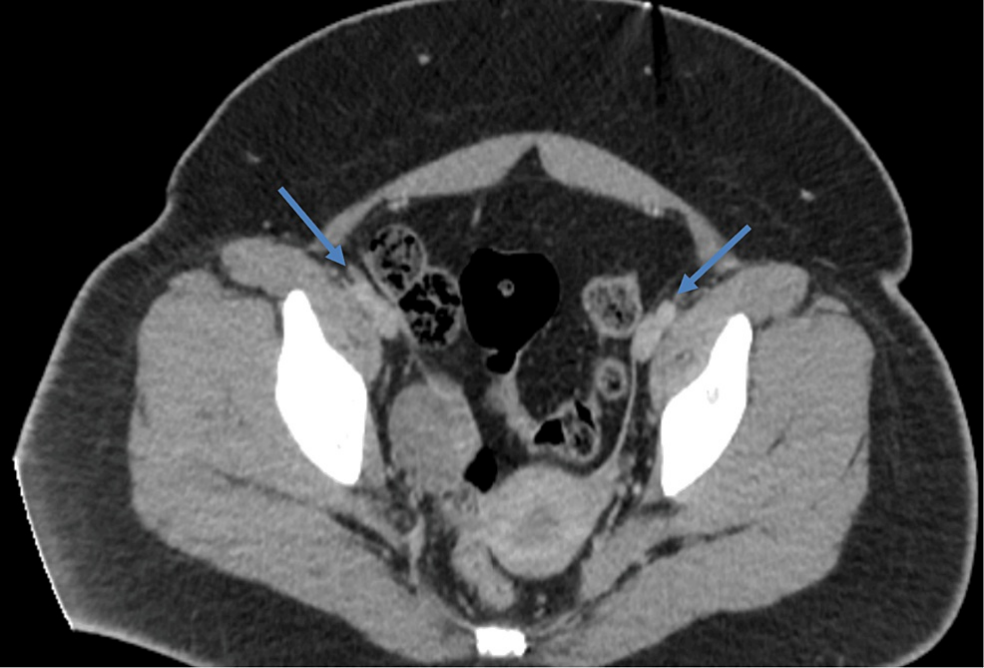
CT of the pelvis negative for pathology Arrows point to ovaries bilaterally. No mass or cyst was noted. CT: computed tomography

Extensive radiographic testing, including pelvic ultrasound (Figure 1) and computed tomography (CT) of the chest (Figure 2), head (Figure 3), abdomen (Figure 4), and pelvis (Figure 5), with intravenous (IV) and oral contrast to evaluate for underlying malignancy, was negative.

For treatment, due to high suspicion for anti-NMDAR encephalitis, the patient was started on high-dose intravenous (IV) Solu-Medrol 1,000 mg daily for seven days, IV immunoglobulin (IVIG) daily for seven days, and plasma exchange (PLEX) for five days, with limited improvement. The patient however demonstrated significant albeit transient clinical and cognitive improvement after a lorazepam trial, suggesting a component of malignant catatonia, which is also a feature of anti-NMDAR encephalitis. She was started on bromocriptine 2.5 mg daily, amantadine 137 mg at night, propranolol 20 mg/12 hours, and topiramate 25 mg daily. Given the clinical constellation of findings, transfer to a tertiary center was initiated for a higher level of care and considerations for electroconvulsive therapy (ECT) and neuroimmunomodulation with Rituxan.

## Discussion

Receptor physiology

**Figure 6:**
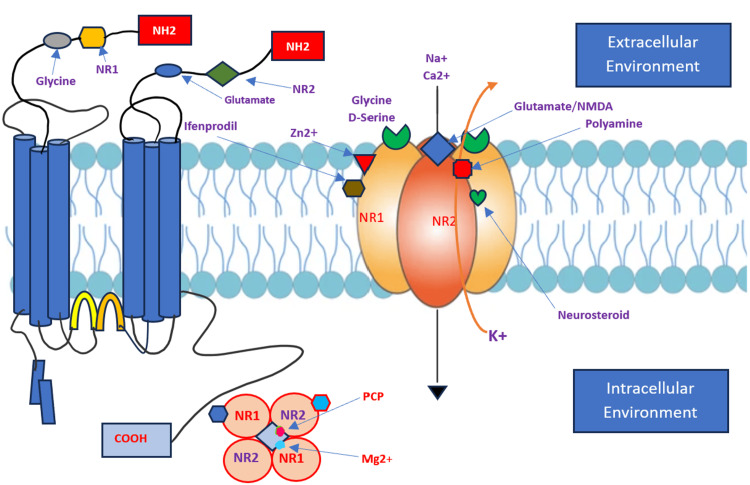
Schematic presentation of the NMDAR structure NMDA has multiple subtypes, which consist of two N1, two N2, and two N3. The N1/N2 complex is the main physiological unit. Activation of the receptor after binding of the ligand closes the NMDA receptor, leading to the opening in the transmembrane ion channel, which is nonspecific for positively charged ions. However, due to the interior chemical properties of the channel, the concentration gradients of ions outside the cell, calcium ions, often pass through the channel. COOH: carboxyl end, NR1: NR1: N-methyl-D-aspartate receptor-1, NR2: NR1: N-methyl-D-aspartate receptor-2, Mg2+: magnesium, PCP: phencyclidine, Na+: sodium, Zn2+: zinc, Ca2+: calcium, K+: potassium Image credit: Ali Ghorbani

N-methyl-D-aspartate receptors (NMDARs) are ion channels involved in memory formation and assist in long-term potentiation. Figure 6 is a schematic presentation of the NMDAR structure. NMDAR subunits NR1 and NR2 are the major functional units of the receptors that assemble to form ligand-gated ion channels containing an agonist recognition site, a transmembrane ion permeation pathway, and gating elements that couple agonist-induced conformational changes to the opening or closing of the permeation pore. These receptors regulate a broad spectrum of processes in the brain, spinal cord, retina, and peripheral nervous system. Glutamate receptors are postulated to play important roles in numerous neurological diseases and have attracted intense scrutiny [4]. The description of glutamate receptor structure, including its transmembrane elements, reveals a complex assembly of multiple semiautonomous extracellular domains linked to a pore-forming element with a striking resemblance to an inverted potassium channel [4]. The functional units of the receptors are arranged as a heterodimer of two NR1 and two NR2 with the central axis running through the entire molecule. These receptors are cation channels, allowing diffusion of positive charges through the cell membrane when activated (Figure 6).

Pathophysiology

Anti-NMDAR encephalitis is an autoimmune disease caused by antibodies against the NR1 or NR2 subunit of the NMDAR [5]. In patients with anti-NMDAR encephalitis, antibodies cause a specific and reversible loss of NMDARs, eliminating NMDAR-mediated functioning of synapses and resulting in associated symptoms [6]. Anti-NMDAR encephalitis is a complicated neuropsychiatric syndrome that can be difficult to differentiate from a purely psychiatric disorder [6]. One study with 571 patients with anti-NMDAR encephalitis found that 4% of patients had isolated psychiatric episodes heralding the initial onset of the disorder. The study found that 74% of patients had delusional thinking, 70% had mood disturbances usually manifesting as mania, and 57% had aggression [7].

Clinical course of the disease

In up to 70% of patients, neurological manifestations are preceded by an infective-like prodrome, characterized by headache, fever, upper respiratory symptoms, nausea, vomiting, and diarrhea. This is followed by symptoms such as hallucinations, agitation, mania, delusions, disorganized thinking, insomnia, and seizures in the following 1-2 weeks. In the months after the initial presentation, patients can expect to develop abnormalities in movement, dysautonomia, hypoventilation, neurological deficits, and seizures. The years following can be marked by impulsivity, disinhibition, disordered executive functioning, and sleep disorders. Often, symptoms improve over time and can even fully resolve [8].

Diagnosis is based on symptoms, electroencephalogram (EEG), and laboratory analysis. Diagnostic criteria are split between probable and definite based on the availability of laboratory and EEG testing. A probable diagnosis of anti-NMDAR encephalitis includes the following three criteria: rapid onset of less than three months of four out of six major groups of symptoms, an abnormal EEG or CSF with oligoclonal bands or pleocytosis, and exclusion of other disorders. Definite diagnosis includes one or more of the major groups of symptoms, anti-NR1 and NR2 IgG antibodies, and exclusion of other disorders (Table 3) [8].

**Table 3:** Diagnostic criteria suggesting possible versus definite anti-NMDAR encephalitis NMDAR: N-methyl-D-aspartate receptor, EEG: electroencephalogram, CSF: cerebrospinal fluid, NR1: N-methyl-D-aspartate receptor-1, IgG: immunoglobulin G Source: [8]

Criteria suggesting possible diagnosis of anti-NMDAR encephalitis
At least four of the following symptoms that started not more than one month ago:
A. Disorder of speech
B. Abnormal cognition or behavior
C. Seizures
D. Movement disorders, dyskinesias, abnormal posturing, or rigidity
E. Decreased level of consciousness
F. Central hypoventilation or autonomic dysfunction
Plus, at least one of the following laboratory results:
A. Abnormal EEG
B. CSF showing oligoclonal bands or pleocytosis
And no other disorder can explain the patient's condition
Criteria suggesting a definite diagnosis of anti-NMDAR encephalitis
One or more of the following symptoms:
A. Abnormal cognition or abnormal behavior
B. Speech dysfunction
C. Seizures
D. Movement disorders, dyskinesias, abnormal posturing, or rigidity
E. Decreased level of consciousness
F. Central hypoventilation or autonomic dysfunction
Plus, presence of anti-NR1 IgG antibodies
And no other disorder can explain the patient's condition

Anti-NMDAR encephalitis laboratory results commonly include CSF findings of elevated white blood cells (WBCs) with lymphocytic pleocytosis, increased CSF protein levels, and the presence of oligoclonal bands. Additionally, serum testing often reveals the presence of antibodies against the NMDAR, confirming the autoimmune nature of the condition [7-9]. Imaging findings in anti-NMDAR encephalitis often demonstrate abnormalities in the hippocampus and amygdala, as observed through magnetic resonance imaging (MRI). These abnormalities may manifest as signal changes, swelling, or atrophy in the affected regions [7].

Two mainstay modalities of treatment for anti-NMDAR encephalitis involve immunotherapy and immunosuppression. Immunotherapy includes the use of corticosteroids, such as Solu-Medrol, IVIG, and PLEX [8]. Corticosteroids play a role in suppressing inflammation, while IVIG provides additional immune modulation. The purpose of PLEX is to remove and replace blood plasma to eliminate pathogenic autoantibodies. Immunosuppressive agents, such as rituximab and cyclophosphamide, are used to prevent the production of autoantibodies [8]. Furthermore, regarding those patients who manifest psychiatric symptoms, including seizures and agitation, benzodiazepines are often used as supportive therapy [9]. The use of benzodiazepines, such as lorazepam and diazepam, is typically for acute symptomatic relief rather than treatment of the underlying autoimmune process [9]. The overall treatment approach, including the incorporation of benzodiazepines, is individualized based on the patient's clinical presentation and response to therapy.

Variable factors such as early treatment, presence of tumors, and severity of symptoms affect the prognosis of anti-NMDAR encephalitis. In a multi-institutional cohort study involving 577 patients, 53% of those who received first-line immunotherapy, such as corticosteroids, IVIG, and/or PLEX, showed improvement within the first four weeks, while those who did not respond and instead received immunosuppressive agents such as rituximab and/or cyclophosphamide had better outcomes than those continuing first-line therapy or no further therapy [10]. While many patients may experience significant recovery, some may continue to face residual cognitive or behavioral challenges [10].

## Conclusions

Our patient who presented with neuropsychiatric manifestations was diagnosed using laboratory workup including CSF studies and serologies, MRI of the brain, and EEG, and thus, first-line treatment was initiated. Additionally, CT of the abdomen and pelvis was obtained, which was negative for malignancy. The patient did return to the ED two months later while on immunosuppressive therapy, back to baseline, and was able to provide her history with complete resolution of the signs and symptoms of the disease. Anti-NMDAR encephalitis has a variable prognosis and requires early recognition with specialty services such as neurology and psychiatry on board, given patients present with diverse symptomatology and may even require an intensive care unit stay. Further studies are needed to evaluate the efficacy of current medical therapy, the role of new immunotherapies, and the need for long-term immunosuppression.
